# Design of a tube lens with a focus tunable lens for optical inspection systems

**DOI:** 10.1038/s41598-026-41904-6

**Published:** 2026-03-11

**Authors:** Younghoon Park, Yong Joo Jo, Jaemyung Ryu, Hojong Choi

**Affiliations:** 1 Department of Optical Engineering, Kumoh National Institute of Technology, Gumi, 39253 Republic of Korea; 2 Optical Solution Division, Samsung Electron-mechanics, Suwon, 16677 Republic of Korea; 3 Department of Electronic Engineering, Gachon University, Seongnam, 13120 Republic of Korea

**Keywords:** Tunable lens, Focus tunable lens, Inspection optical system, Adaptive optics, Imaging and sensing

## Abstract

Optical inspection systems use large numerical apertures (NAs) to achieve high resolution. However, as the NA increases, the depth of field decreases significantly, causing images to blur easily with slight object shifts. Conventional methods maintain focus by mechanically moving the entire lens or specific lens groups. However, these methods increase the system volume, require high-performance motors, and necessitate precise inertial control when the lens group mass is substantial. In this study, we propose a novel tube lens design that integrates a focus tunable lens (FTL), which electronically adjusts its curvature without requiring mechanical movement. We precisely calculated the required curvature radius of the FTL for various object distances and performed optical simulations to verify the system performance before fabrication. The developed tube lens has a focal length of 300 mm and is combined with objective lenses featuring focal lengths of 30 and 60 mm. This enables magnifications of 10X and 5X, respectively. Simulation results confirmed stable image positions for object shifts of ± 50 μm and ± 0.36 mm at 10X and 5X magnifications, respectively, while maintaining diffraction-limited resolution and near-zero distortion aberration. These findings demonstrate that FTL-based focus control is highly suitable for high-precision inspection and imaging applications.

## Introduction

In cutting-edge industries such as semiconductors, displays, and precision part manufacturing, the capability to rapidly and accurately detect microscopic defects or pattern deformations determines the performance and yield of products. The increase in production speed and miniaturization of products hinders the reliable management of quality using conventional visual inspections or simple mechanical methods^1,2^. To overcome these limitations, it is essential to adopt inspection systems with high-resolution optical imaging and automatic defect-detection capabilities^3,4^.

Automated optical inspection integrates key technologies such as visual illumination, high-speed image acquisition, and precise position control to replace human visual inspection and enable fast and accurate detection of defects in real-time production processes^4,5^. In addition, machine vision-based inspection systems contribute significantly to quality improvement and productivity gains through high-performance imaging hardware, advanced image processing, and deep-learning analysis techniques^5,6^. Therefore, inspection optics has been established as a key technology for quality control and productivity improvement in advanced industries. Optical inspection enables rapid and precise defect detection in a non-contact manner and is more reliable and consistent than visual inspection^7^. In particular, automated optical inspection systems minimize the errors caused by worker fatigue and deviations in mass-production environments. Thus, these contribute to improved production efficiency and reduced defect rates through real-time quality monitoring throughout the process^3,6^. In precision part manufacturing industries such as automobiles, aviation, and medical devices, a high level of dimensional and surface quality control is required^8^. High-resolution optical inspection technology based on machine vision is essential for the fast and accurate inspection of parts with complex shapes^6^. These technologies contribute significantly to improving productivity and quality reliability across industries by the early detection of quality variations that may occur in the manufacturing process and support process optimization and the production of high-quality products^9^. In semiconductor manufacturing, the difficulty of defect detection is increasing rapidly because of the high integration and miniaturization of circuit patterns. The defects or undesired substances on the wafer surface directly cause a yield reduction. Therefore, the need for an optical inspection system with high resolution and processing speed is being emphasized further^3,4,10^. The display industry requires high-performance optical inspection to rapidly and accurately detect defects that occur during the production of high-resolution TFT-LCDs and OLED panels^11^. In particular, high-speed non-destructive testing systems can potentially precisely detect even microscopic defects in large-area thin films^12^. Thus, the need for inspecting optical systems capable of high-resolution, high-reliability, and high-speed inspection is growing in various industrial fields.

To obtain high-resolution performance, optical inspection systems generally use a method that adopts large NAs. The NA is directly related to the resolution of the optical system. The larger the NA, the higher the capability to resolve finer structures^13,14^. However, an increased NA results in a reduced depth of field, thereby significantly increasing the focus sensitivity^15,16^. As the depth of field reduces, the focus can shift easily because of external factors such as variations in the height of the sample or micro-vibrations in the system^17^. This, in turn, can reduce the inspection accuracy and reliability. To resolve these problems, technologies that can overcome the limitations of the depth of field or precisely control the focus while maintaining a high resolution are essential for the inspection of optical systems.

Various methods have been developed to control this phenomenon. An inspection system that acquires information from multiple focal planes using chromatic aberration and multifocal plane imaging techniques is proposed^18,19^. In addition, the method of mechanically moving the entire objective lens or a specific lens group is primarily used^20,21^. Alternatively, the microscope stage is moved to alter the position of the sample and focus^22^. This method of focusing by adjusting the position of the lens using a precision stage or actuator is relatively intuitive. It has been widely applied in various optical systems. However, the mechanical drive unit required to move the lens group increases the volume and weight of the system. Moreover, it has limitations in that it results in a complex structure and high cost. Additionally, as the mass of the lens group increases, the response speed decreases. This may cause performance degradation in systems requiring high-speed inspection or real-time focus maintenance. Consequently, new technologies that enable more streamlined and rapid focus adjustments are required.

**Fig. 1:**
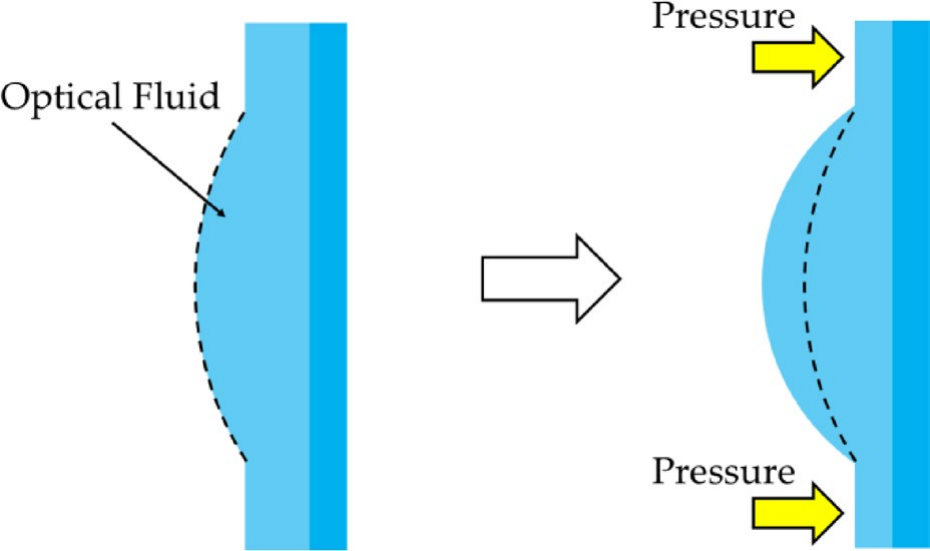
Conceptual overview of FTL operation.

To overcome these limitations, FTLs can be applied. An FTL is based on a structure composed of an optical liquid and an elastic membrane. It adjusts the focus of the lens by modifying the membrane curvature using electrical signals or mechanical pressure^23^. As shown in Fig. 1, the method can alter the focus without moving the lens. This can significantly reduce the volume and weight of the system compared with conventional focus adjustment methods that rely on mechanical drives. It is also suitable for systems that require high response speeds, high-speed inspections, and real-time focused maintenance.

The research on FTL is proceeding actively. Through experimental measurements and theoretical analyses, the surface shape of the FTL can be approximated as a sphere^23–25^. These results indicate that assuming the lens surface to be spherical is reasonable for the design and analysis of FTL. Various FTL structures have been proposed. For example, an FTL that adjusts the curvature of the membrane by redistributing the liquid under an external pressure and, thereby, alters the focal length has been implemented^26^. This method has been indicated to be applicable to various systems based on its simple structure and stable operation characteristics. Furthermore, FTLs employing shape memory alloy actuators to control the membrane curvature have been developed. These provide compact and lightweight designs^27^. In addition, an FTL that alters the focus by adjusting the membrane curvature when using a shape memory alloy spring was implemented^28^. A lens with a large diameter of 34 mm can be focused over a four-diopter range with power consumption below 3 V. In addition, a study was conducted on fabricating a silicon membrane-based FTL and quantitatively analyzing the optical performance according to the structural characteristics when using the modulation transfer function (MTF) and interferometry^29^. Another study was proposed to achieve a high response speed and wide focus adjustment range with a simple structure by integrating a transparent actuator using an electrically active dielectric (dielectric elastomer) into the FTL structure. This method is reported to have a simple optical axis alignment, the capability to adjust the focal length by over two times, and fast response characteristics of the order of tens of milliseconds^30^. Grewe et al. applied FTL to the objective lens of a two-photon microscope for fast axial scanning in 3D biological tissue imaging^31^. However, NA and magnification change when the focus changes, making it unsuitable for industrial inspections that require precise measurements. In our study, NA and magnification variations were maintained within ± 1% of the focus compensation range. Liu and Hua’s research aims to obtain extended depth-of-field images in a single shot^32^. To achieve this, they integrated a liquid lens into the objective lens. However, our designed FTL was integrated into the tube lens. Objective lenses typically have high NAs, making them sensitive to design parameter changes. In contrast, integrating the FTL into the tube lens, which has a relatively low NA and is therefore less sensitive, is more advantageous for maintaining stable performance.

However, in our proposed study, we aimed to resolve the shallow depth-of-field problem of high-resolution optical systems using an FTL. The FTL used is EL-16-40-TC (Optotune, Dietikon, Switzerland)^33^. It has an effective diameter of 16 mm and provides a high response speed of approximately 5 ms. The current allowable range is − 250 mA~+250 mA. The refractive power can be adjusted from − 2 to + 3 diopters. It also has a high transmittance in the wavelength range of 420–950 nm.

In this study, an FTL was integrated into the tube lens of an optical system and placed in a fixed position. The shift of the image plane owing to the variation in the object distance on the objective lens side was compensated through focus adjustment of the FTL. Therefore, an optical inspection system was designed to ensure that the final image sensor surface continuously displayed an image that was in focus. Unlike conventional lens-moving methods, the proposed optical system does not require separate mechanically moving parts. This enables system miniaturization and structural simplification. In addition, it has the advantage of eliminating the need for inertial control owing to lens movement and improving the overall inspection speed by utilizing the high response speed of the FTL. Furthermore, the radius of curvature of the FTL was calculated precisely according to the object distance. These values were used in the optical simulations. This approach enabled performance verification during the simulation stage, thereby improving the design completeness before fabrication.

The remainder of this paper is organized as follows: Sect.  2 outlines the theoretical background of the design of an FTL-based focusing optical system. Section  3 presents the 5X and 10X magnification inspection optical systems utilizing the designed FTL-integrated tube lens with specific objective lenses and reports on their performance analysis conducted through simulation. Finally, Sect.  4 summarizes the research observations.

## Gaussian bracket method for optical systems

A typical optical system comprises multiple surfaces. The larger the number of surfaces, the more complex the equations for ray tracing. To reduce this complexity and express the focal length and main singular points of the optical system more straightforwardly, the Gaussian bracket method was used^34^. The Gaussian bracket method with *n* elements is expressed as Eq. (1):

1 $$\:\left[{a}_{1},\:{a}_{2},\:\cdots\:,\:{a}_{n-1},\:{a}_{n}\right].$$

Gaussian brackets are calculated as in Eq. (2):

2 $$\begin{aligned} \left[ {\:\:} \right] = & 1 \\ \left[ {a_{1} } \right] = & a_{1} \\ [a_{1} ,a_{2} ] = & a_{1} a_{2} + 1 \\ [a_{1} ,a_{2} ,a_{3} ] = [a_{1} ,a_{2} ]a_{3} + \left[ {a_{1} } \right] = & a_{1} a_{2} a_{3} + a_{1} + a_{3} \\ [a_{1} ,a_{2} ,a_{3} ,a_{4} ] = [a_{1} ,a_{2} ,a_{3} ]a_{4} + [a_{1} ,a_{2} ] = & a_{1} a_{2} a_{3} a_{4} + a_{1} a_{2} + a_{1} a_{4} + a_{3} a_{4} + 1 \\ \vdots & \\ \left[ {a_{1} ,a_{2} , \cdots \:,a_{{n - 1}} ,a_{n} } \right] = & \left[ {a_{1} ,a_{2} , \cdots \:,a_{{n - 1}} } \right]a_{n} + \left[ {a_{1} ,a_{2} , \cdots \:,a_{{n - 2}} } \right]. \\ \end{aligned}$$

The Gaussian bracket method is effective for efficiently expressing the results of paraxial ray tracing, which is a ray-tracing method for cases in which the magnitude of the image height is significantly small near the aperture^35^. Paraxial rays are ideal rays that omit aberrations and serve as standards for optical designs^36^.

**Fig. 2:**
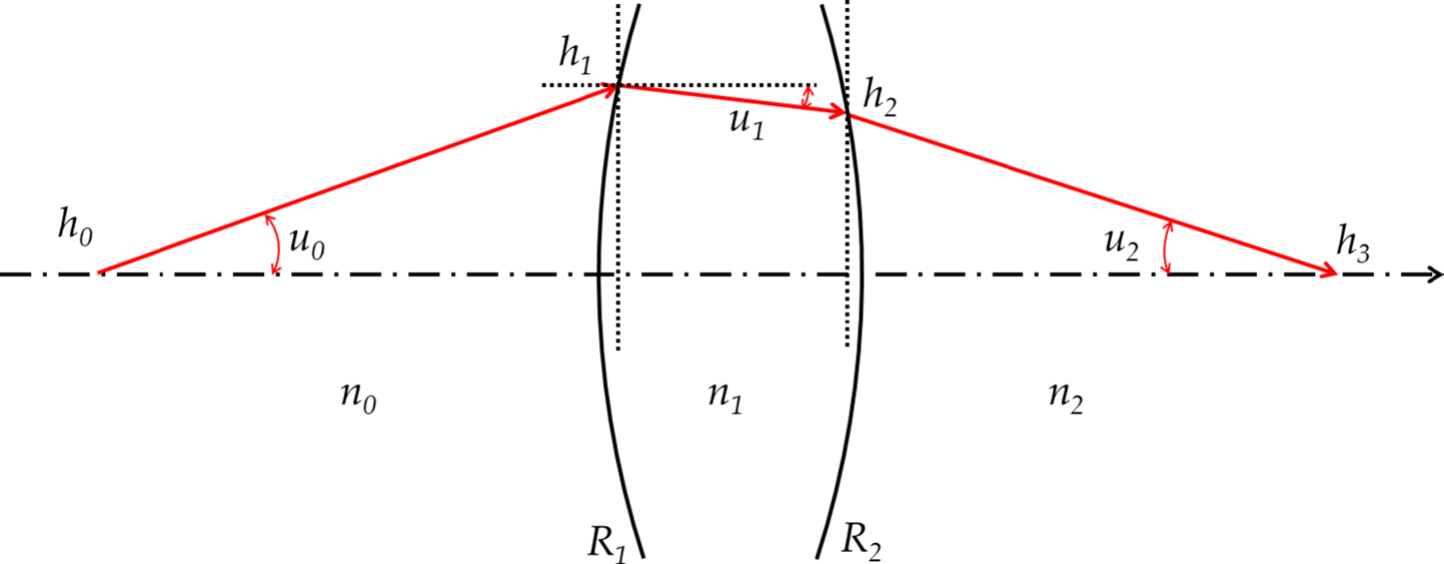
Optical path for the two surfaces.

Figure 2 shows the path of a light ray passing through the two surfaces. *R* is the radius of curvature, *h* is the height of the ray, *u* is the angle of the ray with respect to the optical axis, and *n* is the refractive index. The subscript indicates the number of surfaces.

When a ray passes through an optical system and is refracted or reflected at multiple surfaces, a process is required to track the position and direction of travel of the ray at each surface. Transfer and refraction equations are used for this purpose. The height of the ray at the *i*_*th*_ surface is expressed using the following transfer Eq. (3). The ray angle is expressed by the refraction Eq. (4). In Eq. (5), *k* represents the refractive power of the lens surface.

3 $$\:{h}_{i}={h}_{i-1}+{d}_{i-1}{u}_{i-1}$$

4 $$\:{n}_{i}{u}_{i}={n}_{i-1}{u}_{i-1}-{h}_{i}{k}_{i}$$

5 $$\:{k}_{i}=\frac{{n}_{i}-{n}_{i-1}}{{R}_{i}}$$

Equations (3), (4), and (5) can be expressed in a simple matrix form using the Gaussian bracket method. This is shown in Eq. (6) and is referred to as the system matrix. The ABCD components of the system matrix can be calculated using the Gaussian bracket method and subsequently used to determine the cardinal points of the optical system.

6 $$\begin{aligned} \left[ {\begin{array}{*{20}c} {h_{i} } \\ {n_{i} u_{i} } \\ \end{array} } \right] = & \left[ {\begin{array}{*{20}c} A & { - B} \\ { - C} & D \\ \end{array} } \right]\left[ {\begin{array}{*{20}c} {h_{0} } \\ {n_{0} u_{0} } \\ \end{array} } \right] \\ A \equiv & \left[ {k_{1} , - \frac{{d_{1} }}{{n_{1} }}, \cdots \:,k_{{i - 1}} , - \frac{{d_{{i - 1}} }}{{n_{{i - 1}} }}} \right] \\ B \equiv & \left[ { - \frac{{d_{0} }}{{n_{0} }},k_{1} , - \frac{{d_{1} }}{{n_{1} }}, \cdots \:,k_{{i - 1}} , - \frac{{d_{{i - 1}} }}{{n_{{i - 1}} }}} \right] \\ C \equiv & \left[ {k_{1} , - \frac{{d_{1} }}{{n_{1} }}, \cdots \:, - \frac{{d_{{i - 1}} }}{{n_{{i - 1}} }},k_{i} } \right] \\ D \equiv & \left[ { - \frac{{d_{0} }}{{n_{0} }},k_{1} , - \frac{{d_{1} }}{{n_{1} }}, \cdots \:, - \frac{{d_{{i - 1}} }}{{n_{{i - 1}} }},k_{i} } \right] \\ \end{aligned}$$

The cardinal points of the optical system can be expressed using components ABCD of the system matrix and are calculated using Eqs. (7)–(12). Equation (7) represents the effective focal length (EFL). In this paper, focal length refers to the EFL. Equation (8) represents back focal lengths (BFL). Equation (9) represents the distance from the vertex of the first surface of the optical system to the first principal surface (*H*). Equation (10) represents the distance from the last surface to the second principal surface (*H’*). Equation (11) represents the distance from the first surface vertex to the first nodal point (*N*) of the optical system. Equation (12) represents the distance from the last surface vertex to the second nodal point (*N’*).

7 $$\:EFL=-\frac{{h}_{0}}{{u}_{i}}\:=\frac{{n}_{i}}{C}$$

8 $$\:BFL=-\frac{{h}_{i}}{{u}_{i}}=\frac{{n}_{i}A}{C}$$

9 $$\:H\:=\:\frac{{n}_{0}\left(1-D\right)}{C}$$

10 $$\:{H}^{{\prime\:}}=\:\frac{{n}_{i}\left(1-A\right)}{C}$$

11 $$\:N\:=\:\frac{{n}_{i}-{n}_{0}D}{C}$$

12 $$\:{N}^{{\prime\:}}=\:\frac{{n}_{0}-{n}_{i}A}{C}$$

### Optical modeling of the focus tunable lens

**Fig. 3:**
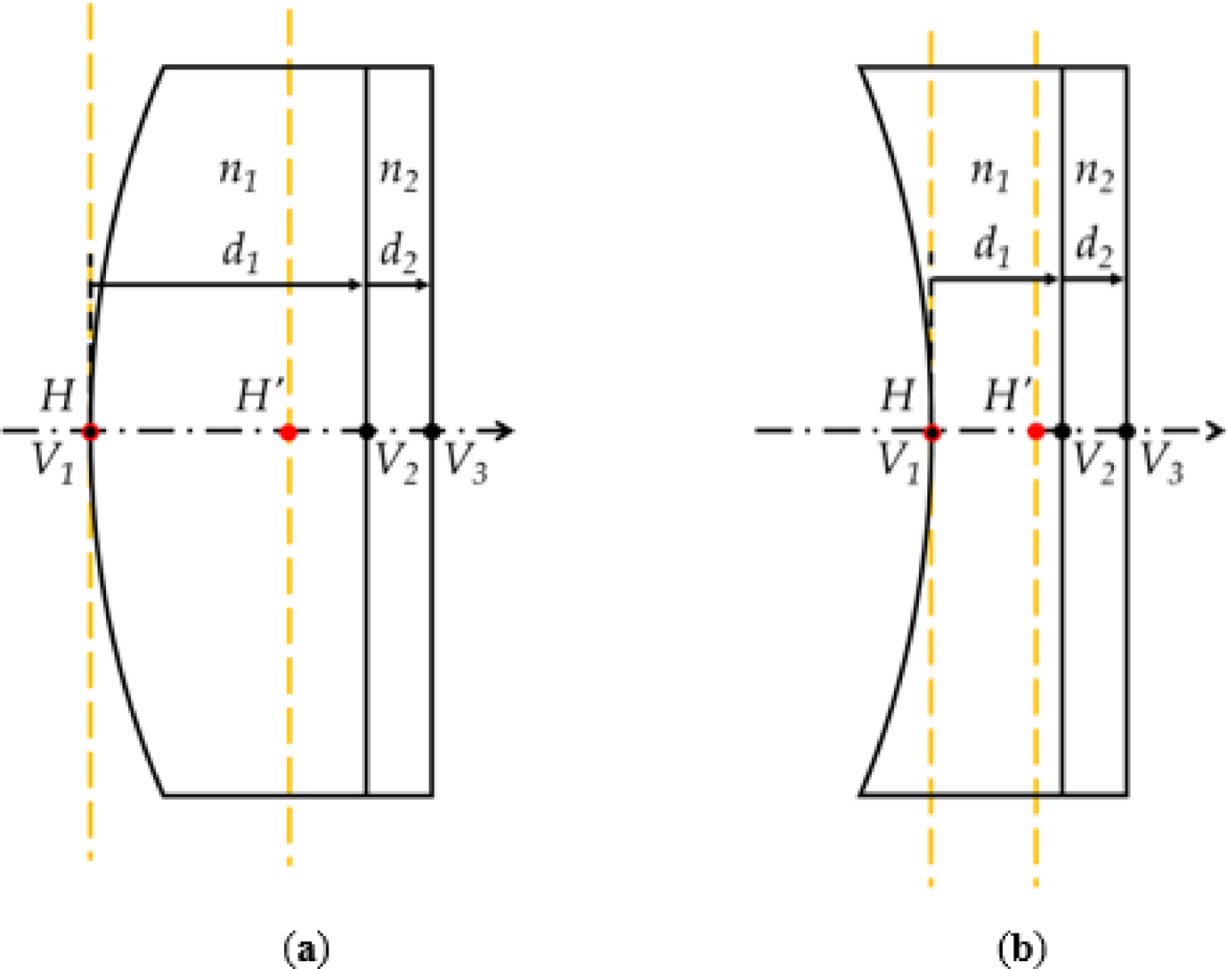
Principal planes of the FTL: (**a**) convex and (**b**) concave lenses.

The FTL used in this study has a flat cover glass placed behind a liquid container. Figure 3 shows the principal planes of an FTL.

*V* is the vertex on each surface, *n* is the refractive index of each surface, and *d* is the distance between the surfaces. *H* and *H’* represent the first and second principal points, respectively. Because the FTL model used here has curvature only on the first surface, the refractive power of the other surfaces is zero. The distance from *H* to *V*_*1*_ is calculated using Eq. (13), and that from *H’* to *V*_*3*_ is calculated using Eq. (14):

13 $$\:H{V}_{1}=0$$

14 $$\:{H}^{{\prime\:}}{V}_{3}=\frac{{d}_{1}}{{n}_{1}}+\frac{{d}_{2}}{{n}_{2}}.$$

### Focus adjustment principles with FTL

**Fig. 4:**
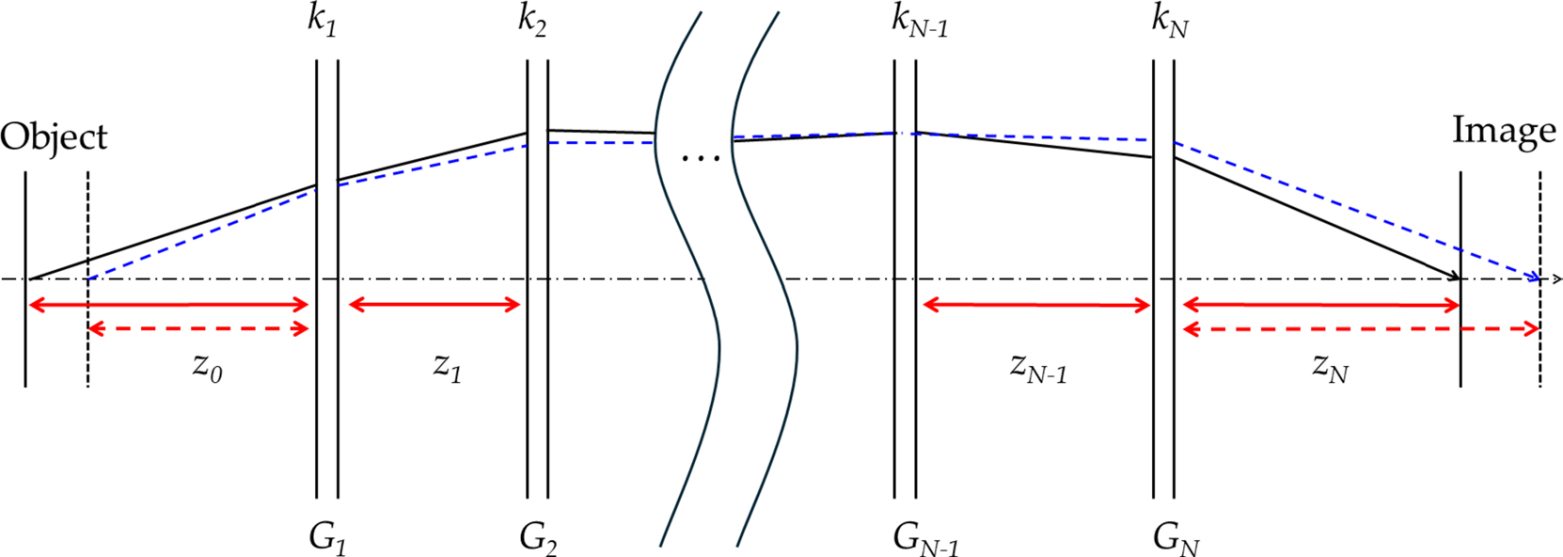
Optical path of an optical system having *N* groups.

In an optical system, a variation in the object distance causes a corresponding shift in the image plane position^37^. This necessitates a focus adjustment to maintain image clarity^38^. Figure 4 illustrates the image plane movement resulting from variations in the object distance.

Conventional optical systems adjust their focus through the mechanical movement of the lens group 39. However, in this study, the focus position was adjusted by the variation in the curvature radius of the FTL. Accordingly, a process was required to determine the optimal curvature radius of the FTL to ensure that the image surface maintained a constant position even when the object distance varied. The zoom equation can be used to calculate the required FTL curvature radius to maintain a fixed image plane position against object distance variation.

Equation (15) describes the transverse magnification of an optical system when the object’s distance is finite. Equation (16) describes the conditions for fixing the image plane when the object distance is finite.

15 $$\:\left[-{z}_{0},\:{k}_{1},\:-{z}_{1},\:\cdots\:,\:-{z}_{N-1},\:{k}_{N}\right]=\frac{1}{\beta\:}$$

16 $$\:\left[-{z}_{0},\:{k}_{1},\:-{z}_{1},\:\cdots\:,\:{k}_{N},\:-{z}_{N}\right]=0$$

*z* is the spacing between the principal planes of each lens group, and *k* is the refractive power of each lens group. *β* indicates the magnification of the entire optical system. The subscripts *z* and *k* indicate the number of the lens group. *z*_*0*_ is the distance from the object to the first principal plane of Group 1, and *z*_*N*_ is that from the second principal plane of Group *N* to the image plane. The zoom equation can be calculated using the Gaussian bracket method 40. The designed optical system consists of three lens groups. The system was implemented by placing the FTL in Group 2, the front lens group in Group 1, and the rear lens group in Group 3.

**Fig. 5:**
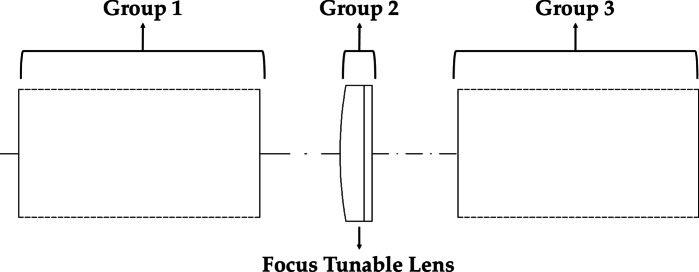
Optical system with three groups, including FTL.

Figure 5 shows a schematic of the three-group optical system in this configuration. In Group 2, a cover glass was placed behind the FTL.

In the optical system, the first and third groups are fixed. Their refractive powers *k*_*1*_ and *k*_*3*_ are constant. Furthermore, the system was designed for a nominal object distance *z*_*0*_ and fixed target image distance *z*_*3*_. In contrast, in the process of focusing using the FTL, the curvature radius of the FTL varies, and the principal planes of the FTL move accordingly. Therefore, *z*_*1*_, *k*_*2*_, and *z*_*2*_ are variables adjusted according to the variation in the curvature radius. When there are three groups, Eq. (16) can be expressed as Eq. (17):

17 $$\:\left[-{z}_{0},\:{k}_{1},\:-{z}_{1},{k}_{2},\:{-z}_{2},\:{k}_{3},\:-{z}_{3}\right]=0.$$

The edge thickness of the FTL remained constant even when the radius of curvature varied, whereas the center thickness varied. Let sag denote the axial distance between the current FTL vertex and its vertex position when flat (infinite radius of curvature). The sag can be calculated using Eq. (18). Here, *R* is the radius of curvature, and *h* is the semi-aperture of the lens.

18 $$sag = ~\left\{ {\begin{array}{*{20}c} {R - \sqrt {R^{2} - h^{2} } ,~R > 0} \\ {R + \sqrt {R^{2} - h^{2} } ,~R < 0} \\ \end{array} } \right.$$

**Fig. 6:**
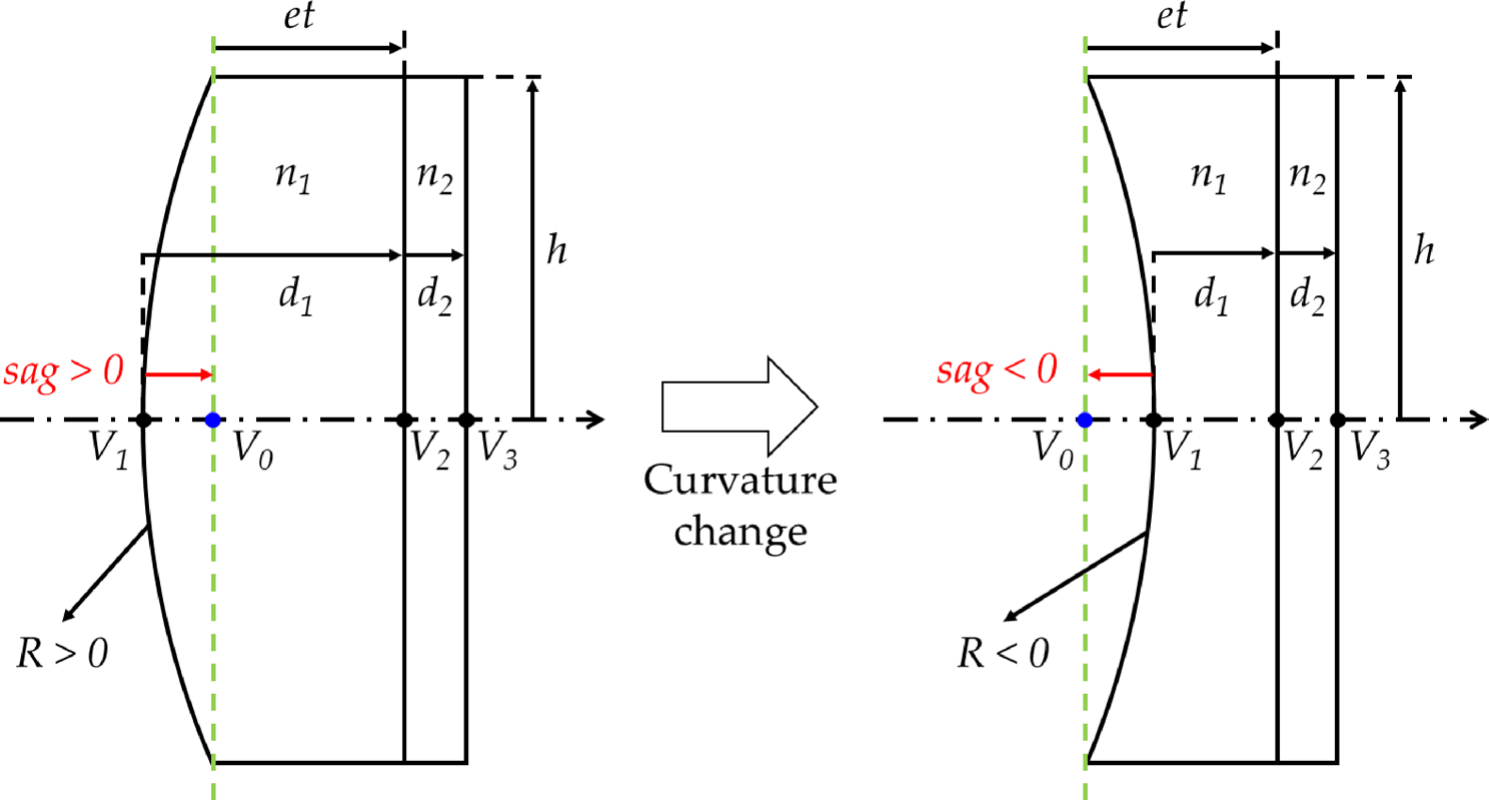
Shape variation owing to FTL curvature variation.

Figure 6 shows the shape variation according to the curvature variation of the FTL.

**Fig. 7:**
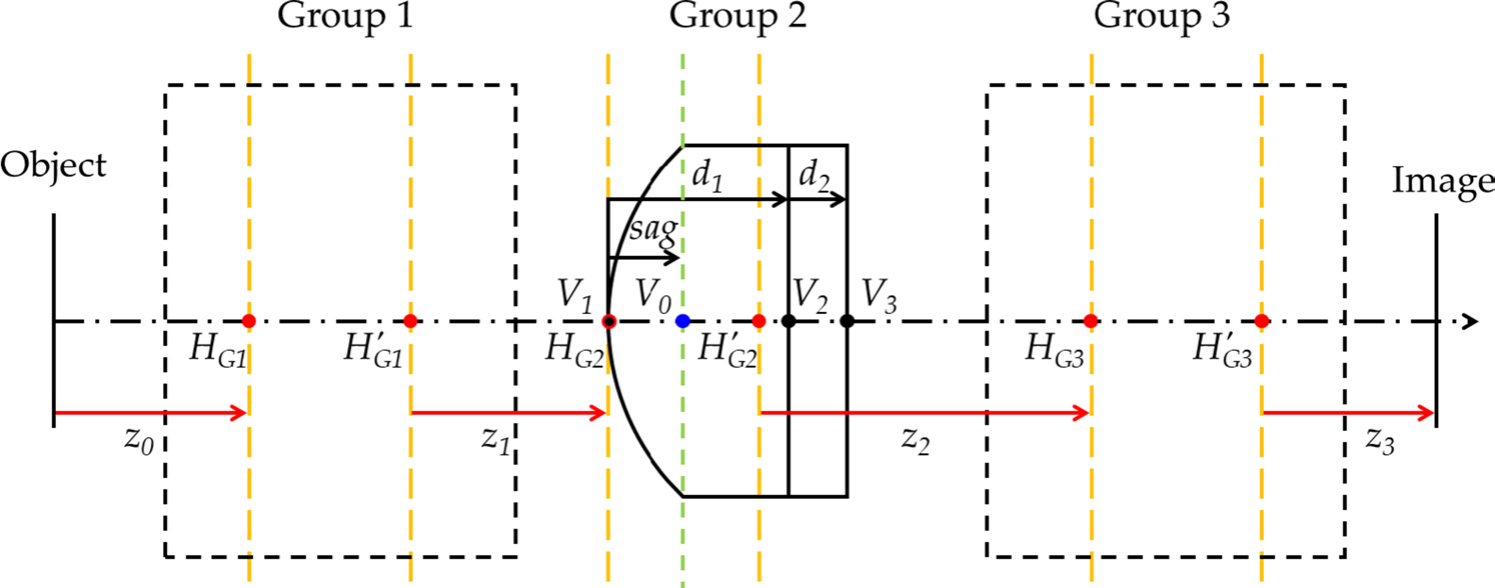
Schematic of the distances between the principal surfaces of an FTL-based three-group optical system.

In Fig. 6, R and *h* represent the radius of curvature of the FTL surface and the semi-aperture of the lens, respectively. *n*_*1*_ and *n*_*2*_ are the refractive indices of the FTL and the cover glass, respectively. *d*_*1*_ and *d*_*2*_ are the central thicknesses of the FTL and cover glass, respectively. *et* represents the edge thickness of the FTL. It remains constant even when *R* varies. *V*_1_, *V*_2_, and *V*_3_ correspond to the vertices of each surface. *V*_*0*_ represents the vertex of the first surface when *R* is infinite. If *sag* is zero, the surface of the FTL is flat, and *d*_*1*_ = *et*. Then, *z*_*1*_ and *z*_*2*_ in Eq. (17) can be expressed in terms of *sag*. Their relationships are shown in Fig. 7.

In Fig. 7, H and *H′* represent the principal points of each group. The principal points of the first and third groups and *V*_0_, *V*_2_, and *V*_3_ have fixed positions regardless of the variation in *R. z*_*1*_ and *z*_*2*_ are calculated as follows:

19 $$\:{z}_{1}={H}_{G1}^{{\prime\:}}{H}_{G2}={H}_{G1}^{{\prime\:}}{V}_{0}-sag$$

20 $$\:{z}_{2}={H}_{G2}^{{\prime\:}}{H}_{G3}={H}_{G2}^{{\prime\:}}{V}_{3}+{V}_{3}{H}_{G3}=\frac{{d}_{1}}{{n}_{1}}+\frac{{d}_{2}}{{n}_{2}}+{V}_{3}{H}_{G3}=\frac{et+sag}{{n}_{1}}+\frac{{d}_{2}}{{n}_{2}}+{V}_{3}{H}_{G3}.$$

*k*_*2*_ is the refractive power of the second group and is expressed as follows:

21 $$\:{k}_{2}=\frac{{n}_{1}-1}{R}.$$

The radius of curvature of the FTL that satisfies Eq. (17) can be determined numerically. By entering the calculated radius of curvature of the variable FTL surface into the optical design program, the image plane can be held constant even as the object distance varies.

### Optical system design and simulation setup

Two objective lenses were used: one with an NA of 0.4 and a focal length of 30 mm, and another with an NA of 0.2 and a focal length of 60 mm. To construct inspection optical systems with 10X and 5X magnifications, each objective lens was combined with a tube lens featuring F/12, a focal length of 300 mm, and an image height of 15 mm. The tube lens incorporates an FTL whose shape can be adjusted to compensate for the image plane shifts resulting from object distance variations when combined with an objective lens. The most characteristic aberration of FTL is gravity-induced coma. This occurs when the weight of the liquid causes the membrane to deform asymmetrically when the optical axis of the lens is horizontal. For this reason, FTL manufacturers recommend that designers use the lens with the optical axis vertical or models with gravity compensation. To optimize the FTL performance, we ado^23^pted the design premise that the optical axis is perpendicular to the ground, which is the typical operating environment for precision inspection equipment. In this condition, the effects of coma aberration due to gravity in FTL are negligible. However, even in an environment where the optical axis is vertical, a slight refractive power change of approximately 0.15 ± 0.01 diopter may occur depending on whether the lens is facing upward or downward. This can be sufficiently compensated by controlling the current applied to the lens when achieving the desired refractive power^33^. The optical system used in this study was designed to operate in the visible light range. It was designed with wavelengths of 656.27, 587.56, 546.07, 486.13, and 435.83 nm, with a center wavelength of 546.07 nm. The optical design software CODE V (Synopsis Inc., East Boothbay, ME, USA, Ver. 2023.03) was used in the design.

### Performance analysis of the 10X system

**Fig. 8:**
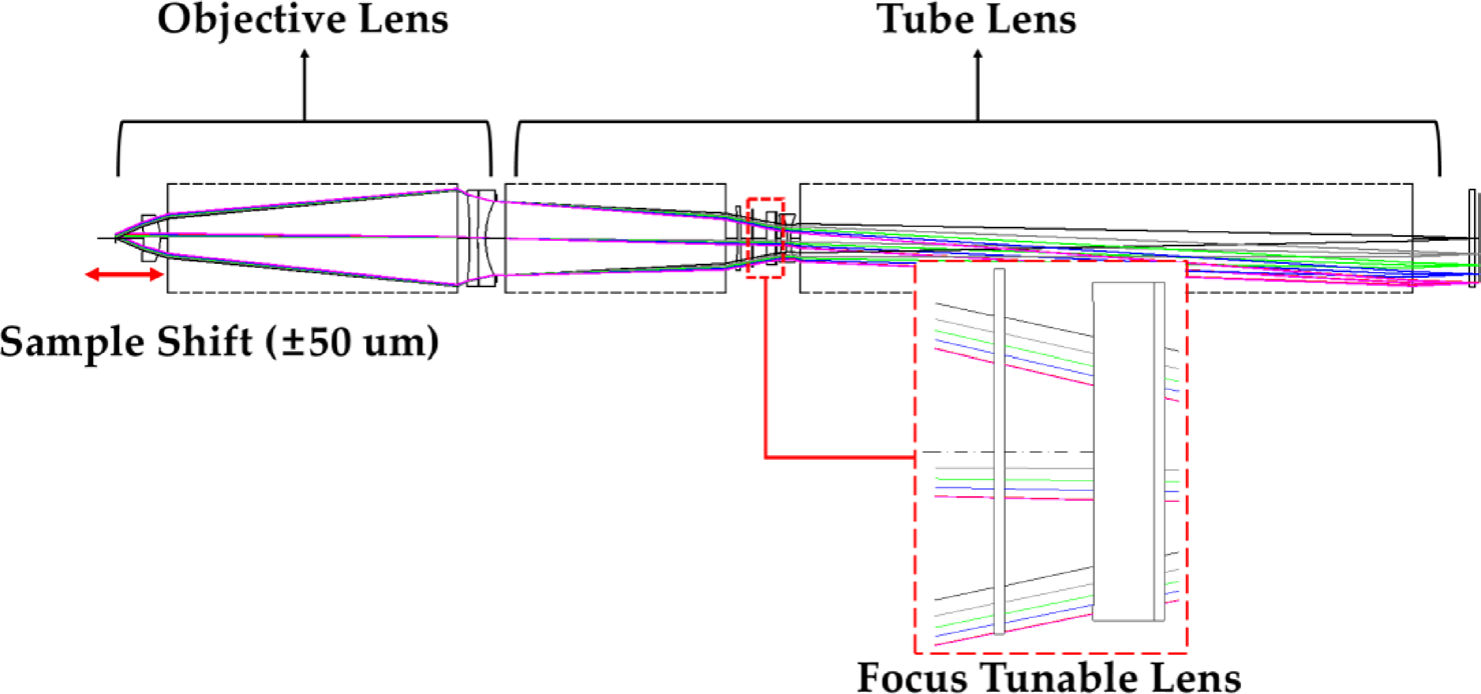
Optical path of the 10X optical system.

Figure 8 shows the optical system at a magnification of 10X. The 10X optical system was designed to achieve a performance close to the diffraction limit for an object distance variation of ± 50 μm relative to the object distance at which the objective lens forms an image at infinity.

The radius of curvature for focusing can be determined using Eq. (17) after modifying the object distance. As shown in Fig. 6, *et* remains constant even as *R* varies. The FTL used in this study has values ​​of *h* = 8.5 mm and *et* = 3.01 mm. Table 1 shows the *R*, *sag*, *d*_*1*_, and *et* of the FTL according to the variation in object distance in the 10X optical system.

**Table 1:** FTL parameters (*R*, *sag*, *d*_*1*_, and *et*) according to the object shift in the 10X optical system.

Object shift (µm)	*R* (mm)	sag (mm)	d_1_ (mm)	et (mm)
–50	–533.104	–0.068	2.942	3.01
0	–1075.184	–0.034	2.976	3.01
+ 50	540708.368	0.000	3.010	3.01

However, because the radius of curvature of the FTL in the tube lens varies, the refractive power of the FTL varies according to Eq. (21). This, in turn, causes a variation in magnification. The magnifications according to the object shift of the optical system are summarized in Table 2.

**Table 2:** Variations in the magnification of the 10X optical system with respect to object shift.

Object shift (µm)	Magnification
–50	10.05
0	9.99
+ 50	9.93

We observed the magnification variation to be approximately ± 0.6% in the 10X optical system. In general, these scale variations may or may not be reasonable depending on the intended use of the system and the precision required. Therefore, whether the variation in scale is within a reasonable range in actual applications should finally be determined based on the user’s criteria and requirements.

**Fig. 9:**
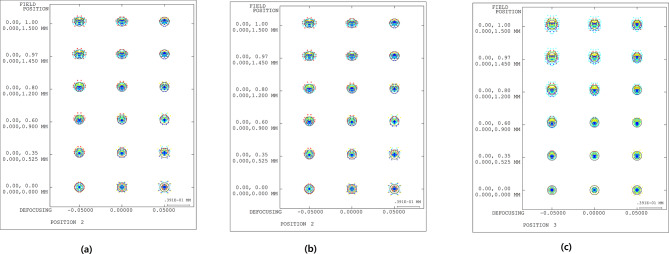
**S**pot diagram of the 10X optical system for **(a)** -50 μm shift, **(b)** reference position, and **(c)** 50 μm shift.

A spot diagram was examined to analyze the performance of the 10X optical system. A spot diagram is a geometric optical point-spread function that depicts the distribution of rays arriving at an image surface; the higher the density of the rays, the better the resolution. Figure 9 shows a spot diagram of the 10X optical system. The x-axis represents the defocus range (analyzed in the ± 0.05 mm range). The y-axis represents the field with object heights between 0 and 1.5 mm.

The circles in the spot diagram represent the Airy disk. The Airy disk is the central bright region of the diffraction pattern formed when plane waves pass through the circular aperture 41. This represents the minimum spot size achievable within the diffraction limit. The size of an Airy disk is expressed as follows 41.

22 $$\:\mathrm{A}\mathrm{i}\mathrm{r}\mathrm{y}\:\mathrm{d}\mathrm{i}\mathrm{s}\mathrm{k}=2.44\frac{f\lambda\:}{D}=2.44\cdot\:\lambda\:\cdot\:(\mathrm{F}/\#)=1.22\frac{\lambda\:}{\mathrm{N}\mathrm{A}}.$$

Here, *λ* represents the wavelength, *f* is the focal length, and *D* is the diameter of the entrance pupil. As indicated in Eq. (22), the size of the Airy disk is proportional to the wavelength and inversely proportional to the NA. Because the image-side NA of the 10X optical system is 0.04, the diameter of the Airy disk at the short wavelength of 435.83 nm was calculated to be 13.29 μm. The resolution was evaluated by comparing the Airy disk and root mean square (RMS) spot size. The RMS spot size represents the average degree of dispersion of the light rays that reach the image plane in the optical system 42,43. The RMS spot sizes at the position where the defocus is zero are summarized in Table 3. For an object distance variation of ± 50 μm, the largest RMS spot size based on the position where the defocus is zero is 12.77 μm. We can verify that it has a small value and high resolution compared with the Airy disk.

**Table 3:** RMS spot size of the 10X optical system when the defocus is zero.

Object height (mm)	(a) RMS (µm)	(b) RMS (µm)	(c) RMS (µm)
1.500	11.77	9.60	12.32
1.450	11.78	9.43	11.84
1.200	11.57	8.83	9.90
0.900	11.94	8.62	8.44
0.525	12.46	8.51	7.45
0.000	12.77	8.50	7.06

**Fig. 10:**
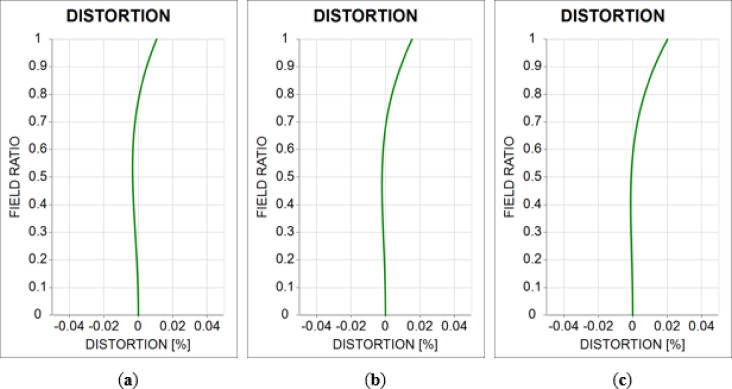
Distortion aberration diagram of the 10X optical system for **(a)** −50 μm shift, **(b)** reference position, and **(c)** 50 μm shift.

Next, we examined the distortion in the 10X optical system. A distortion in the optical system can affect the inspection results. Therefore, an optical inspection system should be designed without distortion. When the positions of the object and image in the optical system vary, the sign of the distortion is reversed. Utilizing this characteristic, the distortion aberration of the tube lens was designed to be comparable to that when the objective lens is reversed in the direction of the optical axis. When the objective and tube lenses are combined, the distortion of each lens is canceled. Figure 10 shows the distortion aberrations of the 10X optical system. The x-axis represents the distortion aberrations. The y-axis represents the field ratio normalized to one over an object height range of 0–1.5 mm.

For a variation in object distance of ± 50 μm, the maximum distortion aberration was 0.02%, confirming a value close to zero. The distortion aberration values ​​of the 10X optical system are summarized in Table 4.

**Table 4:** Distortion aberration of the 10X optical system.

Field ratio	(a) Distortion (%)	(b) Distortion (%)	(c) Distortion (%)
1.0	0.011	0.015	0.020
0.9	0.005	0.009	0.013
0.8	0.001	0.004	0.007
0.7	–0.002	0.000	0.003
0.6	–0.003	–0.001	0.000
0.5	–0.003	–0.002	–0.001
0.4	–0.003	–0.002	–0.001
0.3	–0.002	–-0.001	–0.001
0.2	–0.001	–0.001	–0.001
0.1	0.000	0.000	0.000
0.0	0.000	0.000	0.000

## Performance analysis of the 5X system

**Fig. 11:**
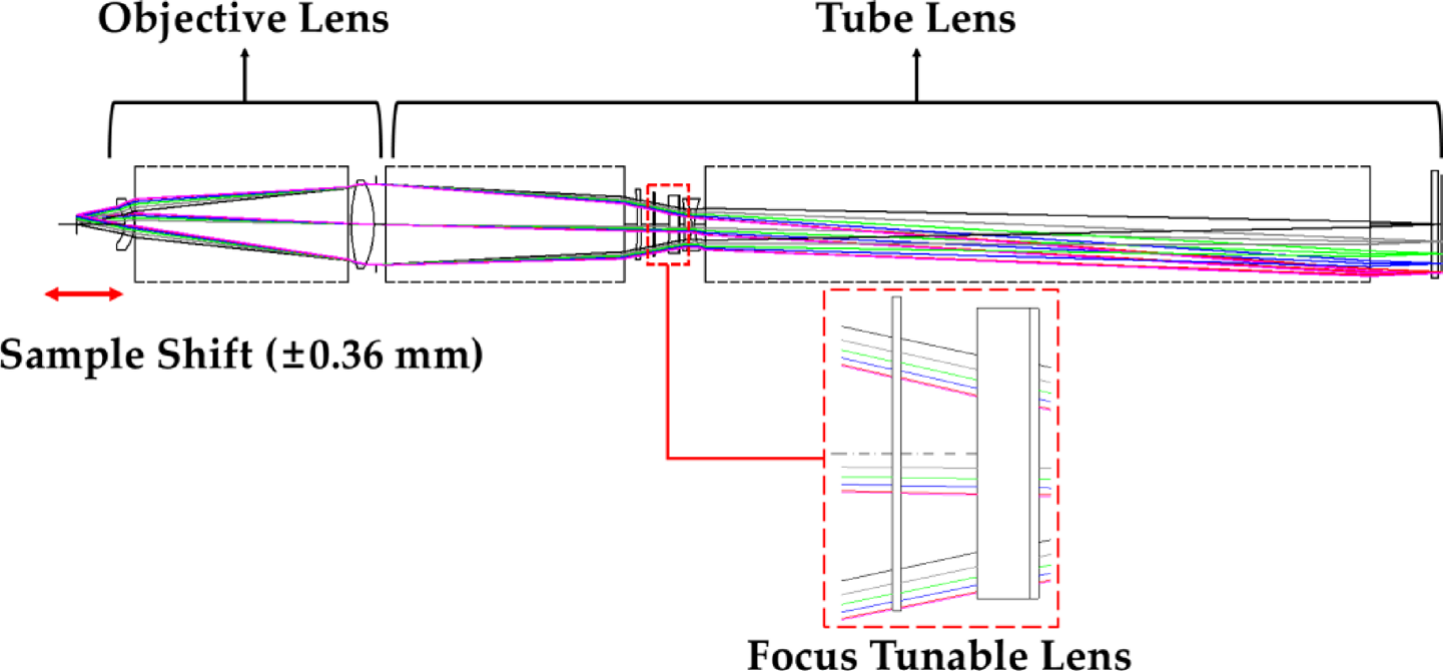
Optical path of the 5X optical system.

To construct an inspection optical system with a magnification of 5X, an objective lens with a focal length of 60 mm and an NA of 0.2 was combined with a tube lens having a 300 mm focal length and an image height of 15 mm. Figure 11 shows the optical path of the system at a magnification of 5X.

Similarly, the 5X optical system was designed to provide a performance close to the diffraction limit for object distances varying by ± 0.36 mm relative to the object distance at which the objective lens forms an image at infinity. Using Eq. (17), we can determine the radius of curvature *R* of the FTL that maintains the image plane fixed for an altered object distance. The *R* and *sag* of the FTL, center thickness *d*_*1*_, and *et* according to the object distance in the 5X optical system are summarized in Table 5. *et* is the value at *h* = 8.5 mm, resulting in a constant value of 3.01 mm regardless of changes in *R*.

**Table 5:** FTL parameters (*R*, *sag*, *d*_*1*_, *et*) according to the object shift in the 5X optical system.

Object shift (mm)	*R* (mm)	sag (mm)	d_1_ (mm)	et (mm)
–0.36	–377.856	–0.096	2.914	3.01
0.00	–1075.184	–0.034	2.976	3.01
+ 0.36	1354.779	0.027	3.037	3.01

Similar to the 10X optical system, the 5X optical system displays variations in the focal length of the tube lens owing to variations in the shape of the FTL. The 5X optical system has a magnification variation of approximately ± 1% for a variation in object distance of ± 0.36 mm. The variation in magnification according to the object distance in the 5X optical system is summarized in Table 6.

**Table 6:** Variations in the magnification of the 5X optical system with respect to object shift.

Object shift (mm)	Magnification
–0.36	5.05
0.00	5.00
+ 0.36	4.95

**Fig. 12:**
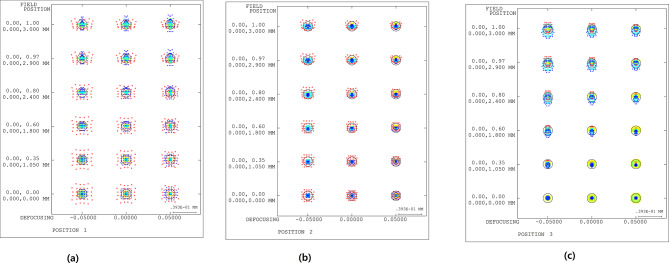
Spot diagram of the 5X optical system for **(a)** −0.36 mm shift, **(b)** reference position, and **(c)** 0.36 mm shift.

To analyze the performance of the 5X optical system, a spot diagram was constructed. Figure 12 shows the spot diagram of a 5X optical system. The x-axis represents the defocus range. The analysis was performed in the ± 0.05 mm section. The y-axis represents the field, with values ​​for object heights ranging from 0 to 3 mm. The 5X optical system has an image-side NA of 0.04. Thus, the Airy disk diameter is 13.29 μm at a short wavelength of 435.83 nm.

For an object distance variation of ± 0.36 mm, the largest RMS spot size at zero defocus is 11.25 μm, which is smaller than the Airy disk diameter. This result indicates that the system maintains a resolution close to the diffraction limit. The RMS spot sizes of the 5X optical system at zero defocus are summarized in Table 7.

**Table 7:** RMS spot size of the 5X optical system when the defocus is zero.

Object height (mm)	(a) RMS (µm)	(b) RMS (µm)	(c) RMS (µm)
3.000	11.25	7.21	10.19
2.900	10.98	6.97	9.91
2.400	9.88	6.40	8.63
1.800	9.97	6.27	7.67
1.050	10.44	6.31	7.43
0.000	10.73	6.35	7.65

Next, we examined the distortion of the 5X optical system. An objective lens with a focal length of 60 mm was designed to have a distortion equal to that of an objective lens with a focal length of 30 mm. Similar to the 10X optical system, the distortion is canceled out when the objective and tube lenses are combined in the 5X optical system.

**Fig. 13:**
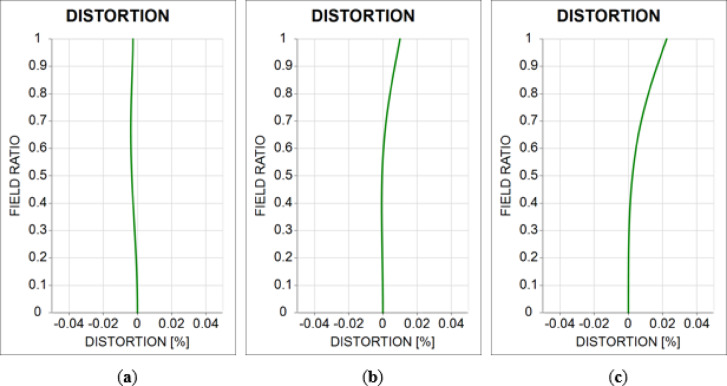
Distortion aberration diagram of the 5X optical system for **(a)** −0.36 mm shift, **(b)** reference position, and **(c)** 0.36 mm shift.

Figure 13 shows the distortion aberration of the 5X optical system. The x-axis represents the distortion aberration. The y-axis represents the field ratio, which was normalized to one based on the object height range of 0–3 mm.

For a variation in object distance of ± 0.36 mm, the maximum distortion aberration was 0.022%, confirming a value close to zero. The distortion aberration values ​​of the 5X optical system are listed in Table 8.

**Table 8:** Distortion of the 5X optical system.

Field ratio	(a) Distortion (%)	(b) Distortion (%)	(c) Distortion (%)
1.0	–0.003	0.010	0.022
0.9	–0.003	0.007	0.017
0.8	–0.004	0.004	0.012
0.7	–0.004	0.002	0.008
0.6	–0.004	0.000	0.004
0.5	–0.003	0.000	0.002
0.4	–0.003	–0.001	0.001
0.3	–0.002	–0.001	0.000
0.2	–0.001	0.000	0.000
0.1	0.000	0.000	0.000
0.0	0.000	0.000	0.000

### Tolerance analysis

**Fig. 14:**
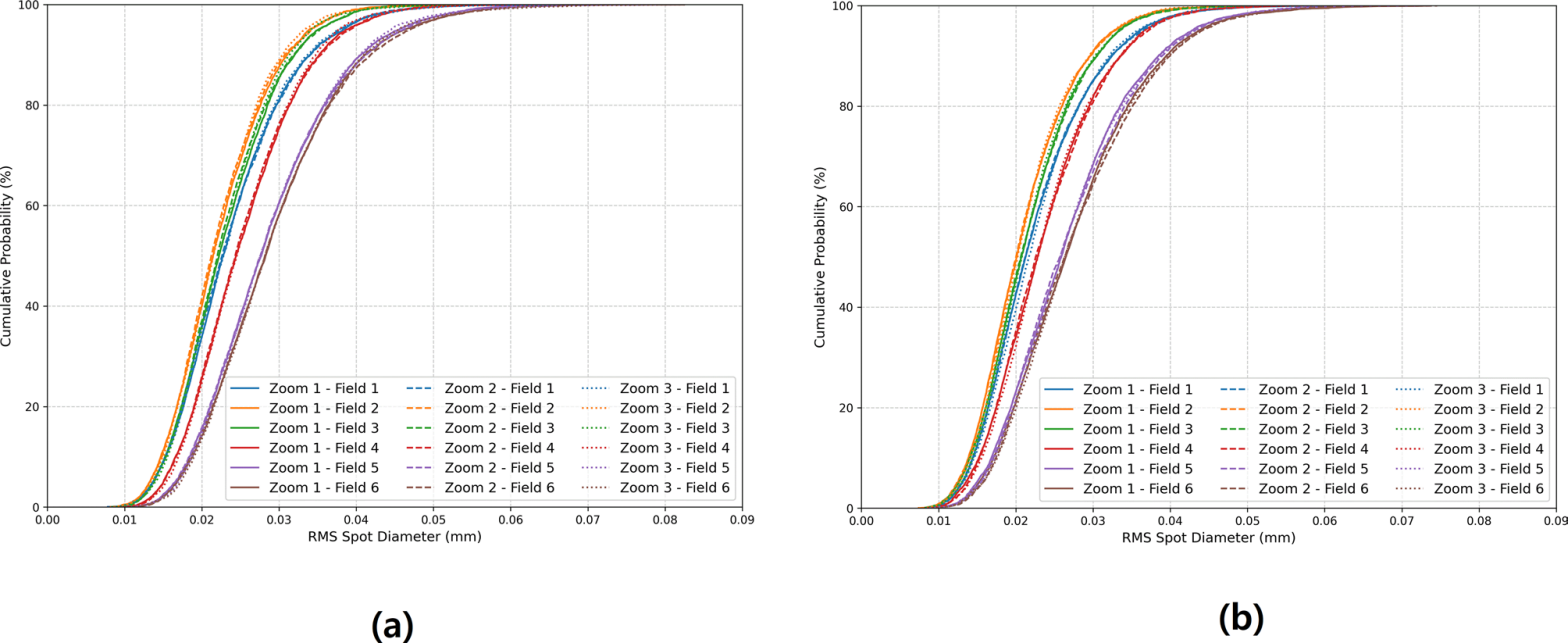
The cumulative probability of RMS spot size for (**a**) 10X and (**b**) 5X optical systems.

The designed optical system is difficult to produce an ideal optical performance during the design stage due to processing and assembly errors. Therefore, considering the manufacturing tolerance, it is essential to predict the production yield that satisfies the required performance during mass production. In our study, 5,000 Monte Carlo simulations were performed using CODE V software for the 10X and 5X optical systems. As tolerance items, the surface sag tolerance was set to ± 1 μm, and the surface irregularity was set to 0.5 fringes. The lens thickness and air gap between parts were set to ± 10 μm, and the material refractive index was set to ± 0.0005. Additionally, for each surface and lens, a decenter error of 5 μm and a tilt error of 3 min were applied, and the same tolerance was applied to FTL to take into account processing and assembly errors. Back focal length was used as a compensator to compensate for the performance degradation. According to tolerance analysis, the final performance of the optical system was evaluated by RMS spot size, and the cumulative probability distribution is shown in Fig. 14. Here, zoom 1, 2, and 3 represent object shifts of − 50 μm, 0 μm, and + 50 μm, respectively, in the 10X system, and − 0.36 mm, 0 mm, and + 0.36 mm, respectively, in the 5X system. The field was defined as the field ratio for six points (0.00, 0.35, 0.60, 0.80, 0.97, and 1.00).

Tables 9 and 10 summarize the tolerance analysis results for the 10X and 5X optical systems, respectively. At the 50% cumulative probability point, the average RMS spot sizes of the 10X and 5X optical systems were predicted to be 24.4 μm and 22.9 μm, respectively, and at the 80% cumulative probability point, they were 31.5 μm and 29.8 μm. Compared to the size of the airy disk, 13.29 μm, the values ​​are approximately 1.8 times larger at 50% and 2.4 times larger at 80% cumulative probability point, suggesting that most systems can maintain stable performance.

**Table 9:** Monte Carlo tolerance analysis data for the 10X optical system.

Cumulative probability	RMS spot size (µm)					
	50%			80%		
Object shift	–50 μm	0 μm	+ 50 μm	–50 μm	0 μm	+ 50 μm
0.00 Field	22.76	22.63	22.81	29.66	29.67	29.45
0.35 Field	21.46	21.23	21.48	27.60	27.33	27.08
0.60 Field	22.24	22.03	22.20	28.50	28.29	28.06
0.80 Field	24.43	24.33	24.53	31.37	31.25	31.13
0.97 Field	27.71	27.57	27.56	35.81	36.03	35.80
1.00 Field	28.27	28.18	28.19	36.44	36.59	36.46

**Table 10:** Monte Carlo tolerance analysis data for the 5X optical system.

Cumulative probability	RMS spot size (µm)					
	50%			80%		
Object shift	–0.36 mm	0 mm	+ 0.36 mm	–0.36 mm	0 mm	+ 0.36 mm
0.00 Field	21.28	21.23	21.70	28.15	28.10	28.08
0.35 Field	20.13	20.03	20.23	25.98	25.98	25.64
0.60 Field	20.73	20.64	20.79	26.60	26.82	26.45
0.80 Field	22.81	22.57	22.84	29.49	29.74	29.18
0.97 Field	25.90	25.71	25.76	33.48	34.13	33.70
1.00 Field	26.47	26.36	26.34	34.48	35.12	34.74

## Conclusion

Optical inspection systems require a large NA to achieve a high resolution. However, as the NA increases, the depth of the field decreases. The theoretical depth of field of the 10X (NA of 0.4) and 5X (NA of 0.2) systems is calculated as *nλ*/NA^2^, which is only about 3.4 μm and 13.7 μm, respectively, based on a central wavelength of 546.07 nm and an object-side refractive index of 1^44^. This causes the image to blur easily, even with a marginal sample movement along the optical axis. Addressing this issue requires a precise focus-control mechanism capable of maintaining an optimal focus notwithstanding variations in the object distance. Conventional optical systems typically achieve focus by mechanically moving the entire lens or a specific group of lenses. However, this method complicates the design of the driving units. Furthermore, the increased mass of the moving lens group necessitates high-performance motors and precise inertial control. This increases the volume and control complexity of the system.

In this study, we integrated an FTL into a tube lens. The proposed method compensates for image plane shifts resulting from object distance variations by adjusting the curvature of the FTL. The FTL has a characteristic of the curvature varying according to the driving current, and can adjust the focus without separate mechanical movement.

The tube lens was designed with F/12, a focal length of 300 mm, and an image height of 15 mm. The FTL was placed at a fixed position within the tube lens. The tube lens was combined with two objective lenses: one with a focal length of 30 mm and an NA of 0.4, and another with a focal length of 60 mm and an NA of 0.2. Therefore, we can implement an optical system at 10X and 5X magnifications. The image position was maintained constant by adjusting the curvature of the FTL for variations in object distance of ± 50 μm and ± 0.36 mm for the 10X and 5X optical systems, respectively. This corresponds to a range that is approximately 29 and 52 times greater than the theoretical depth of field of each system, respectively. In the ideal design, the spot diagram analysis revealed that the RMS spot size remained comparable to that of the Airy disk under all the tested object distance variations, indicating that a resolution close to the diffraction limit was achieved. In addition, the distortion aberration remained close to zero, with a maximum magnitude of 0.022% across the entire range.

Furthermore, the radius of curvature of the FTL was calculated precisely based on the object distance and applied to simulations to improve the completeness of the optical design prior to fabrication. The method proposed in this study provides advantages in terms of system simplification and response speed over conventional mechanical methods, as it enables focus adjustment via electrical control without lens movement. These characteristics are likely to be advantageous for various optical systems, including precision inspection and high-resolution imaging. Furthermore, the versatility of the designed FTL-integrated tube lens enables potential integration with various commercial or custom objective lenses beyond those tested in this study, indicating broader applicability across diverse optical systems.

Our research focuses on proposing new design concepts and rigorously verifying their validity and performance through optical simulations before building an actual prototype for mass production in the industry. Therefore, we used commercial optical design software, which is widely used in the industry. Manufacturing actual optical systems, especially the high-precision system proposed in our study, requires significant time and cost. Therefore, predicting and optimizing performance through simulation during the design phase is crucial and widely practiced in optical system design.

Unlike previous studies that integrated the FTL into the objective lens or used it as a separate component between the objective lens and the tube lens, our study proposes a single module that integrates the FTL into the tube lens. This can simplify complex alignment processes during system assembly and maximize design flexibility and versatility by allowing easy combination with a variety of commercial and custom objectives.

In our study, the influence of gravity was minimized by vertically arranging the optical axis. However, if a large-diameter liquid lens is adopted in the future or the system is expanded in various directions, membrane deformation due to gravity may act as a variable in optical performance. To address these issues, previous studies have proposed technological alternatives that can maintain stable performance without being affected by gravitational deformation even in large-diameter environments^45^. By incorporating these research results into our proposed system, we can expect that the precision inspection applications will be further enhanced in the future.

The optical system in our study suppressed magnification changes to within ± 1% within the focus compensation range (± 50 μm at 10X and ± 0.36 mm at 5X optical systems) and maintained diffraction-limited resolution. This can meet the requirements of industrial inspection equipment that necessitate precise measurements.

## Data Availability

The data used can be made available by the corresponding authors (Jaemyung Ryu and Hojong Choi) uponreasonable request.

## Abbreviations

EFL: Effective focal length
BFL: Back focal length
FTL: Focus tunable lens
RMS: Root mean square
NA: Numerical aperture
